# Social deprivation and secondhand smoke exposure among urban male residents: A nationwide study in China

**DOI:** 10.18332/tid/132290

**Published:** 2021-03-22

**Authors:** Yixin Yang, Xiaozhao Y. Yang, Tingzhong Yang, Wenjiong He, Sihui Peng, Ian R. Rockett

**Affiliations:** 1 School of Public Affairs, Zhejiang University, Hangzhou, China; 2 Center of Social Welfare and Governance, Zhejiang University, Hangzhou, China; 3 School of Sociology and Anthropology, Sun Yat-sen University, Qingdao, China; 4 Center for Tobacco Control Research, Zhejiang University School of Medicine, Hangzhou, China; 5 Department of Health Psychology, Jinan University, Jinan, China; 6 Department of Epidemiology, School of Public Health, West Virginia University, Morgantown, United States; 7 Department of Psychiatry, University of Rochester Medical Center, Rochester, United States

**Keywords:** secondhand smoke, social deprivation, smoke-free policy, public education, health inequities

## Abstract

**INTRODUCTION:**

Social deprivation is a known determinant of health and related behaviors. Many studies have linked socioeconomic factors to secondhand smoke (SHS) exposure. However, no studies have examined the relationship between social deprivation and SHS exposure. This study examined whether contextual social deprivation – variously based on living in a house without a car, that was overcrowded, or had an unemployed member (s) – had an independent association with SHS exposure at both individual and regional levels among Chinese residents.

**METHODS:**

A cross-sectional multistage sampling design was utilized to interview subjects from 6 selected cities in China. A standardized questionnaire selected sociodemographic characteristics, contextual social deprivation and SHS exposure. Multilevel logistic regression models were used to assess the association between social deprivation and SHS exposure.

**RESULTS:**

A total of 5782 valid questionnaires were collected in this study. Among 2930 non-smokers, the SHS exposure prevalence was 21.9% (95% CI: 19.5– 24.30). Multilevel logistic regression showed a negative association between household income, regional GDP, and SHS exposure, respectively, and positive associations between contextual social deprivation and SHS exposure.

**CONCLUSIONS:**

Findings support the central proposition that contextual social deprivation must be factored into SHS exposure messages. Our research underscores the importance of reducing health inequality in controlling SHS exposure.

## INTRODUCTION

Secondhand smoke (SHS), also known as passive smoke, is formed from the burning of cigarettes and other tobacco products and from smoke exhaled by smokers. The World Health Organization (WHO) has estimated approximately one-third of adults and 40% of children worldwide are exposed to SHS^1^. The International Labor Organization estimates at least 200000 workers die each year due to exposure to SHS^2^. An estimated 740 million Chinese are exposed to SHS, including 180 million children aged <15 years^3^. Studies found SHS was very common in households, workplaces and public places^3,4^ in China. According to the 2018 China Global Adult Tobacco Survey conducted by the Chinese Center for Disease Control and Prevention, 50.9% of adults were exposed to SHS in the workplace, 12.9% on public transportation and 73.3% in restaurants. The China Global Youth Tobacco Survey showed that 58.2% of youth (aged 13–15 years) in 2014 were exposed to SHS in outdoor public places, 57.2% in indoor public places, 44.4% at home, and 37.9% on public transportation^3^. Several studies linked exposure to SHS to a number of adverse health consequences in non-smokers, including lung cancer, heart disease, and childhood asthma^5,6^.

Social deprivation is a known determinant of health and related risk behaviors, and associated with multiple diseases^7-9^. People who live in more socially deprived areas have a greater prevalence of behavioral problems than their counterparts in less socially deprived ones^10-12^. Many studies have linked individual-level socioeconomic factors to secondhand smoke (SHS) exposure^13-18^. Disparities increase exposure. Our current study, which was conducted in China, sought new insights into SHS exposure through adding an index of social deprivation, based on living arrangements, as a force that is potentially independent of the impact of the conventional socioeconomic factors of income, education and occupation. This index can be labeled as contextual social deprivation. Examples of relevant elements include living in high density and rental housing, living with persons who are unemployed, and living in a home that lacks a car^19-23^.

## METHODS

### Study type and sampling design

This study was observational, cross-sectional and multilevel, with a multistaged cluster sampling design.

### Study area and participants

Sample cities were selected from across China and differentiated by regional location. Within each city two residential districts were randomly selected from the main urban zones, and four communities were randomly selected within each district. Within each community, the family household registration list was used to randomly sample households. The sample was limited to males aged ≥15 years who had resided in the six study cities for at least 1 year. In the final sampling stage, an eligible person whose birth date was closest to the date of contact was selected from each household to be surveyed if there were two or more male residents. Further details on the sampling are documented elsewhere^24^.

### Data collection

A self-administered questionnaire was scheduled once an individual was identified and agreed to participate in the survey. All responses were anonymous, and each respondent was given an opportunity to seek clarification about survey questions. The same survey protocol was used across the six cities to assure homogeneity of interview and data collection. The survey was administered privately to participants in their home or a designated quiet place, such as a backyard or community park. Surveys were conducted on Saturdays, Sundays, or during the evening, or at other times when the participants were available. The study protocol was approved by the Zhejiang University ethics committee. Verbal consent was obtained from all respondents following instruction from an investigator. Respondents received a gift of 10 RMB (10 Chinese Renminbi about 1.6 US$) after questionnaire completion^24^.

### Variable definition and measurement

#### Dependent variable

SHS exposure was assessed through self-report. We defined SHS exposure as non-smokers who reported daily exposure to SHS for at least 15 minutes per day (Centers for Disease Control and Prevention^5^; Yang et al.^4^). SHS was the dependent variable in this study and was coded dichotomously as: 1=exposure and 0=non-exposure.

#### Independent variable

Our measure or index of contextual social deprivation, based on living arrangements, comprised three elements: residential overcrowding, living in a household without a car, and living in a household with an unemployed member(s)^19-23^. Amount of residential living space was assessed by the question: ‘How many square metres (m^2^) is your home?’. Possible responses were ‘less than 10 m^2^’, ‘from 10 to less than 20 m^2^’, ‘from 20 to less than 30 m^2^’, or ‘more than 30 m^2^’. Residential overcrowding was then measured as family living space per person of less than 10 m^2^. Car ownership was measured through the question: ‘Is there a car in your household?’. Possible responses were ‘no’, ‘one’, ‘two’, or ‘three or more’. The first response was classified as a household without a car. Household unemployment was assessed by a question offering three alternative reponses: ‘no persons’, ‘one’, or ‘two or more’. The second and third reponses signified that a household had an unemployed member(s). Positive categorizations of each of the above kinds of living arrangements were coded dichotomously as: 0=Yes and 1=No. The total score of positive reponses distinguished degree of social deprivation, with values ranging from 0 to 3.

### Covariates

#### Individuallevel independent variables

Sociodemographic characteristics were age, gender, ethnicity, educational level, occupation, and household income. Household income was obtained by asking respondents to report average income per person in their respective households in the previous year. Educational level and household income as well as living arrangements may differentially reflect social deprivation.

#### District in citylevel independent variable

The extent to which people are exposed to SHS could reflect district-level characteristics. Consequently, district economic status was incorporated into this study. It was measured as per capita gross domestic product (GDP), and categorized as <70000, 70000– 99999, and ≥100000 RMB. The data were obtained from the pertinent official local government websites. (http://www.yhtj.gov.cn/upload/file/20170125/6362094967627151571192686.doc).

### Statistical analysis

All data were entered into a database using Microsoft Excel. The dataset was then imported into SAS (9.3 version) for the statistical analyses. Descriptive statistics were calculated for SHS exposure prevalence. Unadjusted logistic models were built for each primary predictor. The multilevel logistic regression model used the SAS NLMIXED procedure to determine associations between contextual social deprivation and SHS exposure^24,25^. We constructed seven models for the multilevel logistic regression analyses. The first was our ‘null’ model, a two-level model (individual and district) with random intercepts. The constant was the sole predictor in accounting for cross-district variation in SHS exposure. In our base model, we entered the conventional sociodemographic variables to form Model 1 (demographic model), as fixed main effects, to evaluate their impact on SHS exposure. Expanding this demographic model, we added household income and our contextual social deprivation variable based on living arrangements, respectively, to form Models 2 (family economic model) and 3 (contextual social deprivation model). We combined these two models to form Model 4 (family economic/contextual social deprivation model). We then entered regional GDP to form Model 5 (regional economic model), and added the contextual social deprivation variable to form Model 6 (regional economic/contextual social deprivation model). SAS 9.3 was applied to run the complex survey data analysis procedure, using the community as the clustering unit in order to account for within-clustering correlation.

All analyses were weighted. Weights included: 1) sampling weights, as the inverse of the probability of selection, calculated at city and district levels, and then multiplied together; 2) non-response weights comprised household and individual aspects; 3) post-stratification weights were calculated using age (<25, 25–34, 35–44, 45–54, and ≥55 years), based on estimated distributions of these characteristics from a national survey^26^. The final overall weights were computed as the product of the prior three sets of weights.

## RESULTS

A total of 6500 individuals were identified as potential participants for this study, of whom 6010 (93.9%) were contacted and agreed to participate in the survey. Of the 6010 surveys collected, 5782 (96.2%) were complete and valid questionnaires. Of the respondents, 2930 were non-smokers and were thus included in this study. Of the sample, 25.4% were aged <25 years, and 23.5% were ≥55 years; most (95.1%) were of Han nationality; 47.6% had high school or less education; approximately 4.8% were divorced or widowers; many worked in operations, were students, or worked in commerce and service occupations (Table 1).

**Table 1:** Demographic characteristics of the sample and secondhand smoke exposure prevalence

Characteristics	*n*	*%*	*Prevalence*	*AOR (95% CI)*
**Age** (years)				
<25	618	25.4	17.9	1.00
25–34	697	19.7	20.0	1.18 (1.02–1.34)*
35–44	618	15.9	22.8	1.37 (0.91–2.07)
45–54	526	15.4	29.8	1.97 (0.96–4.03)
≥55	471	23.5	22.2	1.33 (0.78–2.25)
**Ethnicity**				
Han	2772	95.1	22.1	1.00
Minority	158	4.9	19.3	0.83 (0.53–1.32)
**Education level**				
Elementary school or less	198	10.4	24.7	1.00
Junior high school	462	18.2	28.3	1.21 (1.05–1.39)
High school	709	19.0	25.7	1.06 (0.84–1.33)
Junior college	670	19.1	22.6	0.89 (0.55–1.45)
College or more	891	33.4	15.0	0.52 (0.37–0.71)**
**Marital status**				
Never married	1110	38.1	18.0	1.00
Married	1706	57.1	24.3	1.45 (0.90–2.36)
Divorced	52	1.8	31.9	2.11 (0.59–7.52)
Widowed	62	3.0	17.5	0.96 (0.50–1.86)
**Occupation**				
Manager or clerk	393	10.2	22.9	1.00
Professional	376	12.1	23.0	1.00 (0.74–1.63)
Commerce and service	537	16.0	27.9	1.30 (1.04–1.63)*
Operations	668	20.7	25.5	1.16 (0.87–1.56)
Retiree	270	11.2	16.3	0.66 (0.52–0.84)**
Student	443	19.2	10.8	0.41 (0.31–0.54)**
Unemployed	90	3.0	30.9	1.52 (1.13–2.05)**
Other	161	67	29.2	1.39 (0.89–2.15)
**Household income per capita** (RMB)				
<20000	775	28.8	22.7	1.00
20000–39999	805	27.6	24.5	1.11 (0.97–1.27)
40000–59999	546	16.2	20.5	0.88 (0.61–1.27)
≥60000	804	27.4	19.3	0.82 (0.74–0.90)**
**Contextual social deprivation** (score)				
0	1072	36.6	19.3	1.00
1	1254	45.6	21.1	1.12 (1.02–1.39)*
2	526	19.7	27.7	1.27 (1.15–1.39)**
3	78	2.1	25.3	1.42 (1.08–1.87)*
**District GDP** (RMB)				
<70000	640	8.7	26.4	1.00
70000–99999	1020	34.0	24.6	0.92 (0.78–1.09)
≥100000	1270	59.4	19.6	0.68 (0.54–0.87)**

AOR: adjusted odds ratio. RMB: 1000 Chinese Renminbi about 160 US$.

* p<0.05

** p<0.01.

SHS exposure prevalence varied across age, education, occupation, household and regional income, and degree of contextual social deprivation (Table 1). A multivariable analysis indicated age, occupation, household income, regional income, and contextual social deprivation were all significantly associated with SHS exposure (Table 2). Compared to the reference group, individuals with the highest per capita household income (groups with ≥60000 RMB) had significantly lower SHS exposure prevalence (OR=0.81) than those whose household income was <40000 RMB (Model 2). The groups with contextual social deprivation scores of 2 and 3 had significantly higher SHS exposure prevalence than the group with a score of zero (OR=1.28 and 1.53, respectively) (Model 3). After adjusting for household income, the ORs for contextual social deprivation scores of 2 and 3 were 1.26 and 1.52, respectively (Model 4). In the initial multilevel analysis, persons who lived in the wealthiest districts (GDP per capita ≥100000 RMB) had significantly lower SHS exposure prevalence than the reference group, those living in the poorest districts (OR=0.69) (Model 5). In the second multilevel analysis, following adjustment for the influence of the regional economy, persons with contextual social deprivation scores of 2 and 3 had significantly greater SHS exposure prevalence than those with a score of zero (OR=1.24 and 1.30, respectively) (Model 6).

**Table 2:** Multivariable analysis of secondhand smoking exposure prevalence

*Variables*	*Null model*	*Demographic model (Model 1)*	*Family economic model (Model 2)*	*Contextual social deprivation model (Model 3 )*	*Family economic/contextual social deprivation model (Model 4)*	*Regional economic model (Model 5)*	*Regional economic/contextual social deprivation model (Model 6)*
		*OR (95% CI)*	*OR (95% CI)*	*OR (95% CI)*	*OR (95% CI)*	*OR (95% CI)*	*OR (95% CI)*
**Age** (years)							
<25		1.00	1.00	1.00	1.00	1.00	1.00
25–34		0.82 (0.65–1.03)	0.83 (0.65–1.05.)**	0.83 (0.65–1.06.)	0.54 (0.42–0.78)**	0.56 (0.42–0.74)**	0.55 (0.43–0.70)**
35–44		0.63 (0.42–0.93)*	0.75 (0.56–0.93)*	0.75 (0.55–0.97)*	0.61 (0.49–0.76)**	0.57 (0.47–0.68)**	0.58 (0.49–0.69)**
45–54		0.88 (0.47–1.63)	0.86 (0.64–1.17)	0.86 (0.64–1.17)	0.85 (0.61–1.20)	0.79 (0.53–1.19)	0.81 (0.53–1.21)
≥55		0.75 (0.48–1.18)	0.81 (0.56–1.20)	0.81 (0.56–1.1)	0.71 (0.62–0.82)**	0.69 (0.59–0.81)**	0.71 (0.60–0.84)**
**Occupation**							
Manager or clerk		1.00	1.00	1.00	1.00	1.00	1.00
Professional		0.81 (0.54–1.22)	0.74 (0.36–1.53)	0.74 (0.35–1.54)	0.94 (0.71–1.26)	0.96 (0.72–1.29)	0.96 (0.71–1.30)
Commerce and service		1.00 (0.83–1.19)	1.17 (0.75–1.83)	1.16 (0.75–1.80)	1.17 (0.95–1.45)	1.19 (0.96–1.46)	1.17 (0.95–1.46)
Operations		1.08 (0.82–1.08)	1.19 (0.82–1.73)	1.17 (0.83–1.64)	1.03 (0.81–1.30)	1.05 (0.91–1.21)	1.04 (0.91–1.18)
Retiree		0.43 (0.31–0.59)**	0.69 (0.53–0.90)*	0.69 (0.54–0.89)*	0.59 (0.47–0.74)**	0.59 (0.46–0.77)	0.59 (0.46–0.77)**
Student		0.33 (0.19–0.58)**	0.27 (0.17–0.45)**	0.27 (0.17–0.44)**	0.26 (0.20–0.35)**	0.25 (0.19–0.33)**	0.24 (0.19–0.32)**
Unemployed		0.94 (0.70–1.26)	1.23 (1.01–1.50)*	1.18 (0.91–1.54)	1.02 (0.87–1.22)	1.23 (0.97–1.57)	1.23 (0.95–1.59)
Other		0.89 (0.67–1.19)	1.03 (0.67–1.26)	1.00 (0.66–1.51)	1.29 (1.02–1.66)*	1.19 (0.90–1.56)	1.16 (0.86–1.54)
**Household income per capita** (RMB)							
<20000			1.00		1.00		
20000–39999			1.11 (0.95–1.21)		1.13 (0.98–1.23)		
40000–59999			0.87 (0.63–1.18)		0.87 (0.65–1.22)		
≥60000			0.81 (0.57–0.91)*		0.83 (0.58–0.97)*		
**Contextual social deprivation** (score)							
0				1.00	1.00		1.00
1				1.13 (0.89–1.43)	1.06 (0.91–1.22)		1.09 (0.96–1.24)
2				1.28 (1.09–1.52)**	1.26 (1.07–1.49)**		1.24 (1.09–1.41)**
3				1.53 (1.21–1.83)**	1.52 (1.28–1.81)**		1.30 (1.11–1.76)**
**Per capita GDP in district** (RMB)							
<70000						1.00	1.00
70000–99999						0.94 (0.79–1.12)	0.93 (0.78–1.10)
≥100000						0.69 (0.57–0.81)**	0.68 (0.57–0.80)**
**Fixed parameters**	4.16**	3.20**	2.33*	2.20**	2.8**	3.12**	3.19**
**Random parameters between districts**	3.06**	3.01**	2.88**	2.90**	2.89**	2.35**	2.22*

RMB: 1000 Chinese Renminbi about 160 US$.

* p<0.05

** p<0.01.

## DISCUSSION

This study is the first to examine the association between contextual social deprivation, based on living arrangements, and SHS exposure. We found an SHS exposure prevalence of 21.9% (95% CI: 19.5–24.30) among non-smokers, affirming that SHS exposure among non-smokers is common in China. Socioeconomic inequalities are generally associated with SHS exposure and health problems. Our study also included several conventional individual-level socioeconomic variables, namely, educational level, occupation and household income, together with a regional economic variable, district gross domestic product (GDP) per capita. Occupation and lower educational level were not significantly associated with SHS exposure, findings at variance with those from other studies^3,13,15,17,18,27^. Our anomalous findings may be an artifact of differences in sample demographic composition, since our survey only targeted men. We found that household income and per capita district GDP (regional economy) both manifest negative associations with SHS exposure, affirming findings of many prior studies^4,13,15^. Lower socioeconomic status means fewer educational opportunities, less health awareness, fewer social networks and less safe working environments^28^. In turn, this spectrum of factors may elevate exposure to SHS^4,13^. Lower socioeconomic status also may reflect social deprivation. This study found that household income, regional GDP per capita and contextual social deprivation showed significant independent associations with SHS exposure. However, after we adjusted for the conventional socioeconomic variables and regional economic status, contextual social deprivation still remained in the equation and the odds ratio for household income only changed marginally.

Addressing a gap in the literature, this study found a significant and novel association between contextual social deprivation – variously embodying living in a household without a car, in overcrowded housing and with unemployed member(s) – and SHS exposure. The fundamental cause theory of health argues that health outcomes develop through a series of mediating variables, such as health behaviors, environmental pollution, and risk of injury and harm. Behind these mediators, people can mobilize their flexible resources to avoid harmful mediators and promote beneficial ones^29^. Lacking the full set of resources to modify them, socially deprived individuals are thus inhibited from achieving desirable health outcomes. More importantly, the distribution of these flexible resources, such as schooling, housing, institutional investment, money and connections, are often unequally distributed in a society and subjected to zero-sum competition. The limited total size of flexible resources and their uneven distribution blocks socially deprived individuals from obtaining useful health information and healthcare resources. Personal social deprivation is a powerful determinant of the number and quality of personal health beliefs and behaviors that mitigate the impact of health problems. With fewer available material resources (e.g. money) and symbolic resources (e.g. education and prestige), a person of lower status is likely to be more severely challenged in avoiding risky health behaviors. People with fewer resources have reduced opportunities, less extensive social networks, less personal freedom, more unsafe working conditions, and less confidence in addressing a health threat^30^. Due to subordination in a ranked hierarchy of status and commodities, socially deprived individuals also tend to have less perceived power to control their lives and more suppressed self-efficacy in achieving their goals, which in turn heighten risks of developing mood disorders and other mental illnesses^31^. Although speculative, these same consequences may apply to humans in disadvantaged social positions^32^.

Understanding the nature of the influence of contextual social deprivation on SHS exposure is now crucial in order to reduce the amplifying effect that it exerts upon this exposure, which aligns with other health problems^8,10,11^. The association may be explained by both risky situational exposures and individual resources and behaviors. Individuals with lower socioeconomic status (SES) or in an otherwise socially deprived group may be more likely to smoke^33,34^, and face excess exposure to SHS situations. At the same time, individuals with a socially disadvantaged status tend to lack health awareness, vigilance towards the harmful effects of secondhand smoke, and a behavioral mitigation strategy against secondhand smoke^16,34^, and consequently have a higher prevalence of SHS exposure.

According to the World Health Organization smoke-free policies are the most effective way to reduce exposure to tobacco smoke in public venues^1^. In order to combat the global spread of tobacco use and secondhand tobacco smoke exposure, the WHO established the Framework Convention on Tobacco Control (FCTC) in 1999. Although China lacks a comprehensive smoke-free law, several national laws and policies regulate smoking in public places. Many Chinese cities had instituted smoke-free regulations in public places by the end of 2010^4^. Despite these control measures and regulation, the prevalence of SHS exposure in China remains high. Efforts to restrict smoking in public places should strongly promote the new policies and regulations and fortify the implementation and enforcement process, while simultaneously raising public awareness of the perils of secondhand smoke.

This study provided additional evidence that those living in disadvantaged households and districts had higher SHS exposure rates than those who did not. Our research indicates exposure to secondhand smoke among socially deprived groups is a serious public health problem. Targeted interventions will help reduce health inequities across social classes. While society is changing rapidly, it manifests great social inequality and anomie^35^, which in turn exacerbate health inequality. As Nobel Laureate economist Angus Deaton prudently stated: ‘When inequality is the handmaiden of progress, we make a serious mistake if we look only at average progress. But the story is one of both growth and inequality, not just income, but health too’^36^. Health inequality should be treated as an important social issue. Health inequality was found to exist among individuals of levels of SES and family economic status in China^37^. In 2016, the central government issued the ‘Healthy China 2030’ Planning Outline, which attached great importance to it.

Our study results call for attention to health problems among the disadvantaged. There is an urgent need to address SHS exposure among them in national and local health-sector policies and programs. Public education campaigns need to promote greater awareness of the negative consequences of SHS exposure. Educational efforts especially need to target vulnerable people, with some public awareness campaigns including television, mobile phone media, internet and billboards. Equally important is the implementation of environmental smoking restriction strategies. According to the World Health Organization, smoke-free policies are the most effective way to reduce exposure to tobacco smoke in public venues^38^. There is a tendency for these policies to focus on venues with more favorable conditions for easy implementation of smoke-free measures, such as government offices, hotels, hospitals, schools and public transport. However, this focus ignores the health right of vast numbers of highly vulnerable people to breathe clean air. Although two five-year plans for the Basic Public Service System have been issued in China, none of them covers regulations on SHS control to protect the socially deprived. Smoke-free policies should also cover venues where these people are concentrated, such as construction sites, small factories and sweatshops.

### Strengths and limitations

The study has strengths and limitations. First, an important limitation is the cross-sectional study design, which precludes inference of a causal link between social deprivation and SHS exposure. However, we employed a multilevel study design, both individual and regional, and our findings met several criteria for inferring causality, including the strength of some associations and their consistency. Regardless, it seems implausible that SHS exposure leads to contextual social deprivation, but longitudinal follow-up will provide an opportunity to evaluate this association further. Second, our conception and operationalization of contextual social deprivation was confined to selected living arrangements. In future research, social deprivation could be extended to cover other types of living arrangements, such as whether or not housing was rental and/or multiple, and to include workplaces and frequently visited public spaces. Third, sampling was confined to males. SHS exposure prevalence is very different between males and females, with one study reporting that the prevalence in males is 2.2 times higher than that of females^4^. Hence, the findings of our study cannot be generalized to all. Fourth, there is the possibility of self-report bias in this study because there was no biological verification of SHS exposure.

## CONCLUSIONS

This study provides new information about the influence of social deprivation on exposure to SHS among urban male residents in China. This information may facilitate the understanding of the high SHS exposure prevalence among socially disadvantaged groups there. Our results highlight the need to control secondhand smoke and exposure in order to protect the health of socially and economically vulnerable populations.
